# Signaling molecule glutamic acid initiates the expression of genes related to methylglyoxal scavenging and osmoregulation systems in maize seedlings

**DOI:** 10.1080/15592324.2021.1994257

**Published:** 2021-12-07

**Authors:** Xue-Mei Qiu, Yu-Ying Sun, Zhong-Guang Li

**Affiliations:** School of Life Sciences, Engineering Research Center of Sustainable Development and Utilization of Biomass Energy, Ministry of Education, Key Laboratory of Biomass Energy and Environmental Biotechnology, Yunnan Normal University, Kunming, Yunnan, P.R. China

**Keywords:** Glutamic acid, heat tolerance, maize seedlings, methylglyoxal detoxification system, osmoregulation system

## Abstract

Glutamic acid (Glu) is not only a protein amino acid, but also a signaling molecule, which takes part in various physiological processes in plants. Our previous study found that root-irrigation with Glu could improve the heat tolerance of maize seedlings by plant Glu receptor-like channels-mediated calcium signaling (Protoplasma, 2019; 256:1165–1169), but its molecular mechanism remains unclear. In this study, based on the our previous work, the maize seedlings were treated with 1 mM Glu prior to be exposed to heat stress (HS), and then the expression of genes related to related to methylglyoxal (MG)-scavenging and osmoregulation systems was quantified. The results showed that Glu treatment up-regulated the gene expression of *Zea mays* aldo-keto reductase (*ZmAKR*) under both non-HS and HS conditions. Also, the gene expression of *Zea mays* alkenal/alkenone reductase (*ZmAAR*), glyoxalase II (*ZmGly II*), pyrroline-5-carboxylate synthase (*ZmP5CS*), betaine dehydrogenase (*ZmBADH*), and trehalase (*ZmTRE*) was up-regualted by exogenous Glu treatment under HS conditions. These data imply that signaling molecule Glu initiated the expression of genes related to MG-scavenging and osmoregulation systems in maize seedlings, further supporting the fact that Glu-enhanced heat tolerance in plants.

Glutamic acid (Glu) is not only a protein-building block, but also a signaling molecule, which regulates various plant physiological processes in plants.^1^ Glu takes part in seed germination, root architecture, pollen germination, and pollen tube growth, as well as plant response to environmental stresses.^2–5^ Our previous study also found that Glu enhanced the heat tolerance by coupling calcium signaling system in maize seedlings, but the underlying molecular mechanisms remain unclear.^6^

Methylglyoxal (MG), similar to Glu, has dual role: cellular toxic agent and signaling molecule.^7^ Therefore, MG must be maintained homeostasis in plant cells by its scavenging system composed of aldo-keto reductase (AKR), alkenal/alkenone reductase (AAR), glyoxalase I (Gly I), and glyoxalase II (Gly II), corresponding key genes are *ZmAKR1, AmAAR1, ZmGly I*, and *ZmGly II* in maize.^8^ Under HS conditions, MG can be over-accumulated, causing MG stress (similar to oxidative stress), that is, proteins, nucleic acids, and biomembrane damage. Meanwhile, HS can trigger osmotic stress (i.e. water deficiency), which can be alleviated by osmoregulation system.^9,10^ Osmoregulation system is involved in the osmolytes (e.g. proline, glycine betaine, trehalose, and total soluble sugar) and their key metabolic enzymes (pyrroline-5-carboxylate synthase: P5CS; betaine aldehyde dehydrogenase: BADH; trehalose-5-phosphate phosphatase: TPP; and trehalase) in plants, corresponding key genes are *ZmP5CS, ZmBADH, ZmTPP*, and *ZmTRE1* in maize.^11^ Therefore, MG-scavenging and osmoregulation systems play a key role in the formation of plant heat tolerance.

To further explore the effect of Glu treatment on the expression of genes related to MG-scavenging and osmoregulation systems in maize seedlings, based on our previous study,^6^ the maize seedlings were irrigated with 1 mM Glu for 6 h, and then HS 16 h. Experiments were performed three biological repeats, and data was tested using one-way analysis of variance (ANOVA) and the least significant difference (LSD). The gene expression of *ZmAKR1, ZmAAR1, ZmGly I, ZmGly II, ZmP5CS, ZmTPP, ZmBADH*, and *ZmTRE1* was quantified by qRT-PCR (using *Zea mays* beta-5 tubulin (*ZmTUB*) as reference gene) in maize seedlings under both Glu treatment and HS conditions. The results displayed that under non-HS Glu treatment significantly increased the expression of *ZmAKR1* (at 6 h), while significant difference did not observed in *ZmAAR1, ZmGly I, ZmGly II, ZmP5CS, ZmTPP, ZmBADH*, and *ZmTRE1* (Figure 1). Under HS conditions, treatment with Glu improved the gene expression of *ZmAKR1* (at 8 h), *ZmAAR1* (at 16 h), *ZmGly II* (at 16 h), *ZmP5CS* (at 16 h), *ZmBADH* (at 16 h), and *ZmTRE1* (at 8 h) (Figure 1). For *ZmGly I* and *ZmP5CS*, Glu treatment did not have effect on their expression (Figure 1). These results suggest that signaling molecule Glu initiated the expression of genes related to MG-scavenging and osmoregulation systems in maize seedlings under both non-HS and HS conditions, further supporting our previous study that Glu enhanced heat tolerance in maize seedlings. In addition, the results of gene expression also further imply that MG-scavenging and osmoregulation systems play a vital role in the development of heat tolerance in plants.

**Figure 1.**
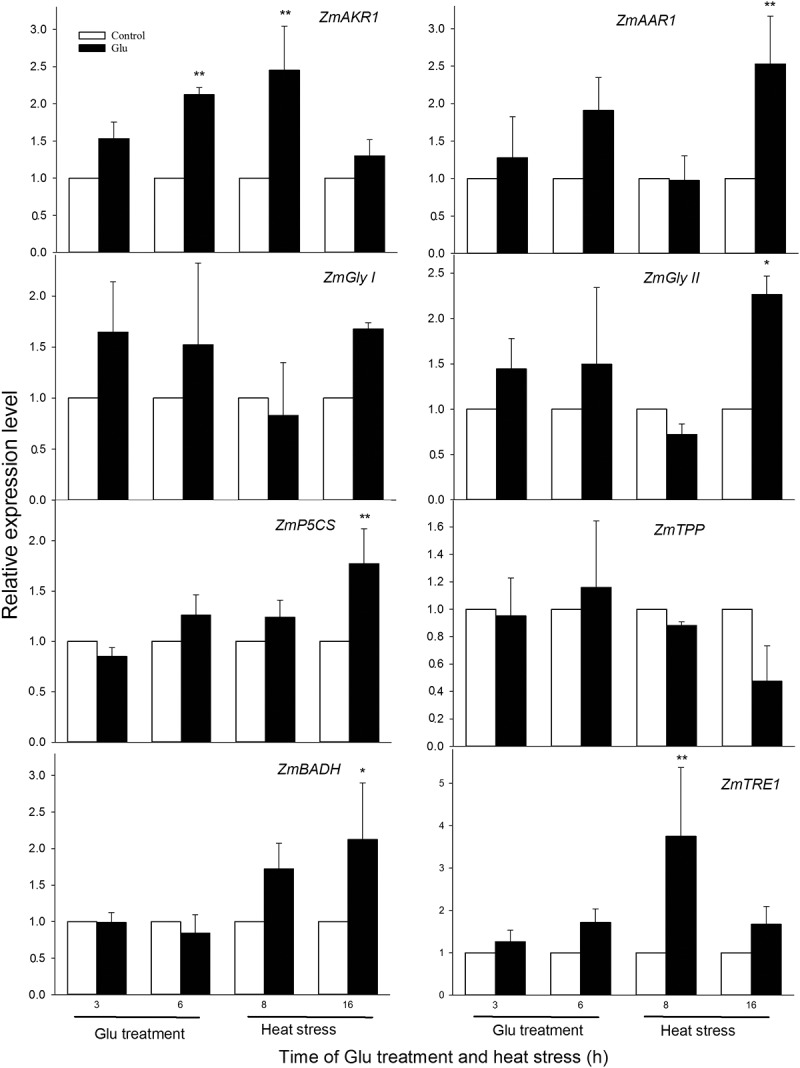
Effect of glutamic acid (Glu) on the gene expression of *Zea mays* aldo-keto reductase (*ZmAKR*), alkenal/alkenone reductase (*ZmAAR*), glyoxalase I (*ZmGly I*), glyoxalase II (*ZmGly II*), pyrroline-5-carboxylate synthase (*ZmP5CS*), trehalose-6-phosphate phosphatase (*ZmTPP*), betaine dehydrogenase (*ZmBADH*), and trehalase (*ZmTRE*) in maize seedlings under non-HS and HS conditions. Relative expression level was expressed using the ratio of experimental group to control group, which represents the mean ± SE (n = 3), and asterisk (*) and double asterisks (**) indicate significant difference (*P* < .05) and very significant difference (*P* < .01) compared with the control without Glu treatment, respectively.
